# A historical controlled study of domestic trastuzumab and pertuzumab in combination with docetaxel for the neoadjuvant treatment of early HER2-positive breast cancer

**DOI:** 10.3389/fonc.2024.1281643

**Published:** 2024-02-09

**Authors:** Dongdong Xu, Jiang Wu, Jing Yu, Yuqing Yang, Xinxin Wen, Jixin Yang, Hongliang Wei, Xiaolong Xu, Yike Li, Liu Yang, Lei Wang, Yijia Wang, Wen Ma, Nanlin Li

**Affiliations:** ^1^ Department of Thyroid Breast & Vascular Surgery, Xi’jing Hospital, Air Force Medical University (AFMU), Xi’an, China; ^2^ Department of Psychology, Colorado College, Colorado Springs, CO, United States; ^3^ Department of Medical Oncology, Dana-Farber Cancer Institute, Harvard Medical School, Boston, MA, United States; ^4^ Center for Functional Cancer Epigenetics, Dana-Farber Cancer Institute, Boston, MA, United States

**Keywords:** trastuzumab, pertuzumab, docetaxel, neoadjuvant treatment, HER2-positive breast cancer

## Abstract

**Background:**

HER2-positive molecular breast cancer subtypes are characterized by high aggressiveness and malignancy, and their metastasis and mortality rates are among the highest of all types of breast cancer. The use of anti-HER2-targeted agents in neoadjuvant therapy has significantly improved the prognosis of patients with HER2-positive breast cancer. In this study, we investigated the efficacy and safety of a neoadjuvant Chinese THP regimen (docetaxel, trastuzumab biosimilar TQB211 plus the pertuzumab biosimilar TQB2440 or pertuzumab) for ER/PR-negative and HER2-positive breast cancer in China.

**Method:**

All enrolled patients received the THP regimen (T: docetaxel 75 mg/m2 per cycle; H: trastuzumab biosimilar TQB211 8 mg/kg in the first cycle and 6 mg/kg maintenance dose in cycles 2 to 4; P: pertuzumab biosimilar TQB2440 or pertuzumab 840 mg in the first cycle, maintenance dose 420 mg in cycles 2 to 4) every 3 weeks for 4 cycles. The biosimilar TQB2440 pertuzumab and pertuzumab were randomly assigned to patients. Docetaxel, TQB211, and TQB2440 were all developed by Chiatai Tianqing. The primary endpoint was the complete pathological response (pCR) in the breast, and the secondary endpoint was cardiac safety.

**Results:**

Of the 28 eligible patients, 19 (67.9%) achieved tpCR. The tpCR rate was higher than in the NeoSphere trial (pCR63.2%) and the PEONY study (tpCR52.5%). The adverse events that occurred most frequently were leukopenia and neutropenia, with incidence rates of 82.1% and 75.0%, respectively. Of these, grade 3 leukopenia and neutropenia occupied 46.4% and 35.7%. Other grade 3 or higher adverse events were bone marrow suppression (7.1%), lymphopenia (3.6%), and anemia (3.6%). There were no events of heart failure in patients and no patient died during the neoadjuvant phase.

**Conclusion:**

Domestic dual-target HP has a more satisfactory efficacy and safety in the neoadjuvant phase of treatment.

**Clinical trial registration:**

https://clinicaltrials.gov/study/NCT05985187, NCT05985187.

## 1 Introduction

Today, breast cancer is the most common cancer in women and the leading cause of death from cancer worldwide (1). Human epidermal growth factor receptor-2 (HER2) is overexpressed in 15–25% of breast cancer patients, and HER2-positive molecular subtypes are aggressive and extremely malignant. HER2-positive breast cancer had the highest metastatic rate and mortality rate of all breast cancer (2). With the wider application of monoclonal antibodies, dual-antibodies, TKI, and ADC drugs, the prognosis of HER2 positive breast cancer has improved considerably (3–6), and approaches that of the luminal classification (7). Neoadjuvant therapy (NAT) has greatly improved tumor resection and breast conservation rates, and pathologic complete remission (pCR) serves as a reliable prognostic indicator: achieving pCR results in longer survival (8). Therefore, neoadjuvant therapy has become the standard of care for locally advanced breast cancer and is increasingly being used in the treatment of early-stage breast cancer (9, 10). The use of anti-HER2 targeted agents in NAT regimens has significantly improved the prognosis of patients with HER2-positive breast cancer (3, 11–13). Trastuzumab is a humanized recombinant anti-HER2 monoclonal antibody that binds to the extracellular region of HER2 with high affinity and specificity, producing antitumor effects by blocking the HER2 signaling pathway (2). Trastuzumab was the first therapeutic agent targeting HER2, and was initially approved for the treatment of advanced HER2-positive breast cancer. Given the favorable results of clinical trials, the use of trastuzumab increased alongside its indication for the treatment of early-stage breast cancer (4, 5). Numerous studies have shown that inhibition of HER2 alone with trastuzumab in conjunction with chemotherapy has a lower therapeutic efficacy than neoadjuvant dual-HER2 blockade using pertuzumab and trastuzumab (14, 15). This made dual-targeting plus chemotherapy the standard neoadjuvant treatment strategy for high-risk HER2-positive breast cancer (16, 17). Dual-targeting plus chemotherapy is more effective in treating HER2-positive breast cancer, according to the NeoSphere and PEONY studies. However, there are few reports on clinical studies involving domestic dual-targeted medications in China, whereas recent large clinical trials have evaluated dual-targeted drugs manufactured by foreign companies. Therefore, we investigated dual target plus chemotherapy for HER2-positive breast cancer in China to address the gap in this field.

## 2 Patients and methods

### 2.1 Patients and study design

The objective of this study was to compare the efficacy and safety of a domestic dual-target therapy (trastuzumab biosimilar TQB211+ pertuzumab biosimilar-TQB2440) with that of foreign dual-target drugs (trastuzumab + pertuzumab) drugs. The domestic dual-target pre-stage clinical trial used enrollment criteria and protocols similar to those of the NeoSphere and PEONY studies.

All enrolled patients met the following criteria: (i) age: 18–75 years; ECOG PS score: 0–1; (ii) patients with pathologically histologically or cytologically confirmed primary breast cancer; (iii) met the American Cancer Society (AJCC) 8th edition TNM stage of breast cancer II - III Stage C (T2-T4 plus any N, or any T plus N1-3, M0), with locally advanced, inflammatory or early stage, unilateral and histologically-confirmed invasive breast cancer; (iv) HER2 positivity confirmed by central laboratory; (v) patients confirmed negativity for both estrogen receptors (ER) and progesterone (PR); and (vi) patients who agreed to undergo mastectomy after neoadjuvant therapy to meet the surgical criteria. The exclusion criteria were as follows: (i) patients with stage metastatic breast cancer or patients who, in the judgment of the investigator, could not undergo radical surgical resection with neoadjuvant therapy; (ii) patients with bilateral invasive breast cancer; (iii) patients who had received previous chemotherapy, endocrine therapy, or anti-HER2 biologic therapy; (iv) patients with other malignancies appearing within 3 years or that are currently concurrent; (v) patients with male breast cancer, and (vi) patients with uncontrolled hypertension or impaired cardiac function (LVEF <55%).

The neoadjuvant regimen was the THP regimen. where T was docetaxel, H was the biosimilar trastuzumab TQB211, and P was the biosimilar pertuzumab TQB2440 or pertuzumab (Paget®). A total of 28 patients were enrolled. All patients received the same T and H. TQB2440 and pertuzumab were randomly assigned 1:1 to the 28 patients. Drugs were administered as follows:

(i) TQB2440/pertuzumab: intravenous infusion, 1 treatment cycle of 3 weeks, administered on the first day of each cycle, for a total of 4 cycles, with a loading dose of 840 mg in cycle 1 and a maintenance dose of 420 mg in cycles 2 to 4.
(ii) TQB211: intravenous infusion, 1 treatment cycle of 3 weeks, administered on day 1 of each cycle. A total of 4 cycles were used, with a loading dose of 8 mg/kg in cycle 1 and a maintenance dose of 6 mg/kg in cycles 2 to 4.
(iii) docetaxel: intravenous infusion, 1 treatment cycle of 3 weeks, each cycle administered at a dose of 75 mg/m^2^, used for a total of 4 cycles.

Docetaxel, TQB2440 and TQB211 were produced and developed by the CHIATAI TIANQING Company.

All patients underwent surgery after 4 cycles of neoadjuvant therapy. During adjuvant treatment, we provided including 3 cycles of FEC regimen and 13 cycles of dual target therapy.

The primary endpoint was complete pathological response. Surgery was performed within 4 weeks of completion of the 12-week neoadjuvant therapy, and tpCR and bpCR were evaluated by the pathology laboratory at Xijing Hospital. tpCR was defined as noninvasive tumor cells on microscopic examination of breast and axilla specimens after primary tumor excision (ypT0/is ypN0), and bpCR was defined by the presence of noninvasive tumor cells on microscopic examination of the breast after primary tumor excision (ypT0/is).

The secondary endpoint was patient safety outcomes during treatment.

During neoadjuvant treatment, every 3 weeks, a physical examination, physical status of the ECOG, routine blood counts, blood biochemistry, coagulation, routine urine, routine stool, ECG test, and echocardiogram were completed in each cycle of follow-up. Imaging was performed after every cycle, and breast MRI was performed at the end of cycle 4. Before the first cycle of neoadjuvant therapy, patients underwent various evaluations to identify distant metastases through the use of mammography, breast, and abdominal ultrasound, chest and head computed tomography, bone scan, breast magnetic resonance imaging (MRI), and blood tests. Continued monitoring for adverse events and serious adverse events was performed until 21 days after the last treatment. Ongoing monitoring and classification of adverse events were performed according to the National Cancer Institute Common Terminology Criteria for Adverse Events Version 3.0.

All procedures involving human participants in this study were performed in accordance with the Declaration of Helsinki (revised 2013). The retrospective study protocol was approved by the Ethics Committee of the Xijing Hospital, a hospital affiliated with the Air Military Medical University. All patients provided informed consent for their data to be used for the purpose of the study.

### 2.2 Data analysis and statistical methods

This study was a historical controlled study to compare effectiveness and safety with that of previously available neoadjuvant clinical trials. SAS 9.2 (SAS Institute Inc., USA) was used for statistical analysis. For data analysis, tpCR/bpCR was expressed as a rate, clinical and pathological characteristics of the patients were expressed as frequencies (percentages), adverse effects during treatment were expressed as frequencies (percentages), and the left ventricular ejection fraction (LVEF) was expressed as a rate. The graphing software used was R v.4.2.1.

## 3 Results

**Table 1** summarizes the baseline clinical and pathological characteristics of the 28 patients in our investigation.

**Table 1:** Baseline clinical and pathological characteristics of the 28 patients enrolled in the study.

Age	
>50 years old	16 (57.1%)
≤50 years old	12 (42.9%)
BMI	
<18.5	1 (3.6%)
18.5–23.9	16 (57.1%)
>23.9	11 (39.3%)
Menstrual status	
Premenopausal	15 (53.6%)
Postmenopausal	13 (46.4%)
Location	
Left side	16 (57.1%)
Right side	12 (42.9%)
Puncture HER2	
+2 and FISH determined 3	1 (3.6%)
+3	27(96.4)
Puncture Ki-67 expression	
>30%	18 (64.3)
≤30%	10 (35.7%)
Histological grading	
II	20 (71.4%)
III	8 (28.6%)
Clinical staging	
IIA	9 (32.1%)
IIB	11 (39.2%)
IIIA	5 (17.9%)
IIIB	1 (3.6%)
IIIC	2 (7.1%)
cT staging	
T1	
T2	20 (71.4%)
T3	5 (17.9%)
T4	2 (7.1%)
cN Staging	
N0	10 (35.7%)
N1	15 (53.6%)
N2	1 (3.6%)
N3	2 (7.1%)

A total of 28 ER/PR-negative, HER2-positive breast cancer patients with a mean age of 49 years were enrolled in this study, all of whom had invasive breast cancer (nonspecific type). Two patients had a family history of cancer, in one case a sister with esophageal cancer and in the other case a mother with rectal cancer. **Table 1** shows the baseline clinical and pathological characteristics of the 28 patients in our investigation.

After four cycles of neoadjuvant chemotherapy regimens, all patients underwent radical mastectomy for breast cancer. After postoperative pathology evaluation, 19 of the 28 patients achieved tpCR, with a tpCR rate of 67.9%. In contrast, patients in the PEONY study who received the neoadjuvant chemotherapy PTH*4 regimen achieved a tpCR rate of 39.3% postoperatively as determined by an independent review committee, while those with ER/PR-negative, HER2-positive breast cancer achieved a tpCR rate of 52.5% (17). In contrast, the results of the NeoSphere study showed that the pCR rate achieved in breast cancer patients was 63.2% (16), those with ER/PR-negative, HER2-positive breast cancer in a chemotherapy regimen of trastuzumab combined with docetaxel on top of a dual target of trastuzumab in NAT. The tpCR rate in this trial was higher than that in the ER/PR-negative, HER2-positive group in the PEONY and NeoSphere studies that also received dual targeting combined with docetaxel.

Each patient experienced at least one adverse event potentially related to the study treatment. **Table 2** describes the most common adverse events during the neoadjuvant period, and **Table 3** reports the grade 3 and higher adverse events and comparisons. Leukopenia and neutropenia were the most common adverse events, with incidences of 82.1% and 75.0%, respectively, and both were also responsible for the most frequent incidence of grade 3 and higher adverse events of 46.4% and 35.7%, respectively. A serious adverse event occurred during treatment: febrile neutropenia. The patient resolved with symptomatic treatment and the event did not affect subsequent treatment. No discontinuation of treatment due to drug-related adverse events occurred during the course of treatment.

**Table 2:** Incidence of the most common adverse events during neoadjuvant therapy trials across the three THP regimens studies.

Our Study		PEONY Study		NeoSphere Study	
Leukopenia	23 (82.1%)	Hair loss	107 (49.1%)	Hair loss	68 (64%)
Neutropenia	21 (75.0%)	Neutropenia	105 (48.2%)	Neutropenia	54 (50%)
Hair loss	19 (67.9%)	Leukopenia	92 (42.2%)	Diarrhea	49 (46%)
Diarrhea	18 (64.3%)	Diarrhea	84 (38.5%)	Disgusting	41 (38%)
Mouth ulcers	13 (46.4%)	Anemia	53 (24.3%)	Fatigue	28 (26%)
Rash	9 (32.1%)	Elevated alanine aminotransferase	49 (22.5%)	Rash	28 (26%)
Lack of power	6 (21.4%)	Disgusting	45 (20.6%)	Mucosal inflammation	28 (26%)
Constipation	6 (21.4%)	Elevated aspartate aminotransferase	37 (17.0%)	Myalgia	24 (22%)
Fever	5 (17.9%)	Fever	31 (14.2%)	Asthma	22 (21%)
vomiting	5 (17.9%)	Upper respiratory tract infection	33 (15.1%)	Headaches	12 (11%)

**Table 3:** Incidence of grade 3 and higher adverse events for the three studies during the neoadjuvant period.

This study		PEONY Study		NeoSphere Study	
Leukopenia	13 (46.4%)	Neutropenia	83 (38.1)	Neutropenia	48 (45%)
Neutropenia	10 (35.7%)	Leukopenia	45 (20.6)	Febrile neutropenia	9 (8%)
Bone marrow suppression	2 (7.1%)			Leukopenia	5 (5%)
Lymphocytopenia	1 (3.6%)			Diarrhea	6 (6%)
Anemia	1 (3.6%)			Asthma	2 (2%)
Serious adverse events		Serious adverse events		Serious adverse events	
Febrile neutropenia	1 (3.6%)	Febrile neutropenia	4 (1.8%)	Neutropenia	4 (4%)
				Febrile neutropenia	6 (6%)

The most common grade 3 or 4 adverse reaction in the PEONY study was neutropenia, with an incidence of 38.7% (17), and the incidence of neutropenia among grade 3, 4 and above adverse reactions in this study was 35.7%, which was essentially the same as the incidence in PEONY and slightly lower than the 45% incidence of neutropenia among grade 3 and 4 adverse reactions in the NeoSphere study (group receiving the THP neoadjuvant chemotherapy regimen), which was slightly lower (16). The incidence of leukopenia among grade 3, 4 and above adverse reactions in this study was 46.4%, which is twice as high as the 20.6% (leukopenia incidence) in PENOY (17) and higher than the incidence in the THP group in NeoSphere (5%) (16).

**Figure 1:**
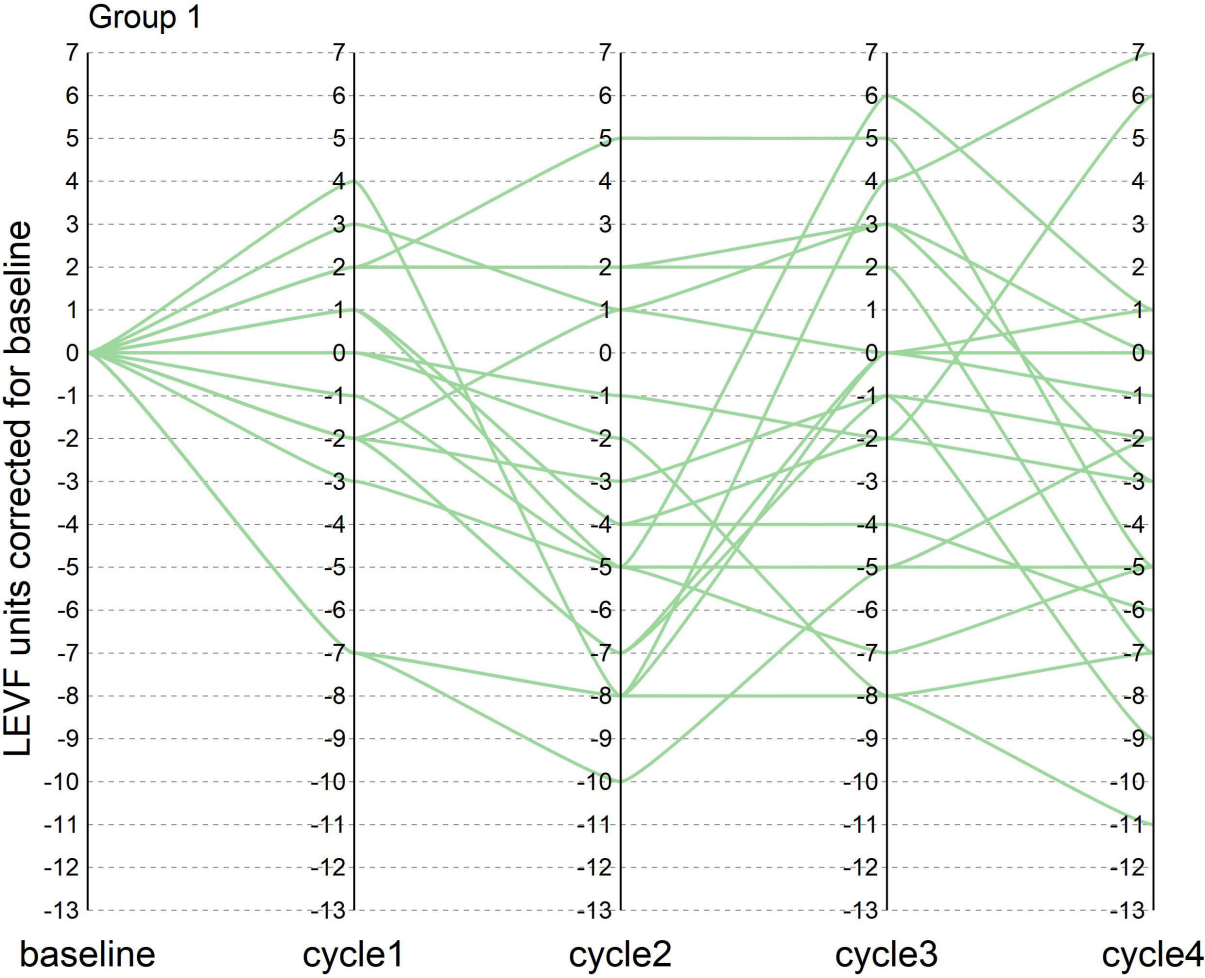
**Figure 1** shows the change in LVEF compared to baseline in patients who reached tpCR during the treatment in our research.

**Figure 2:**
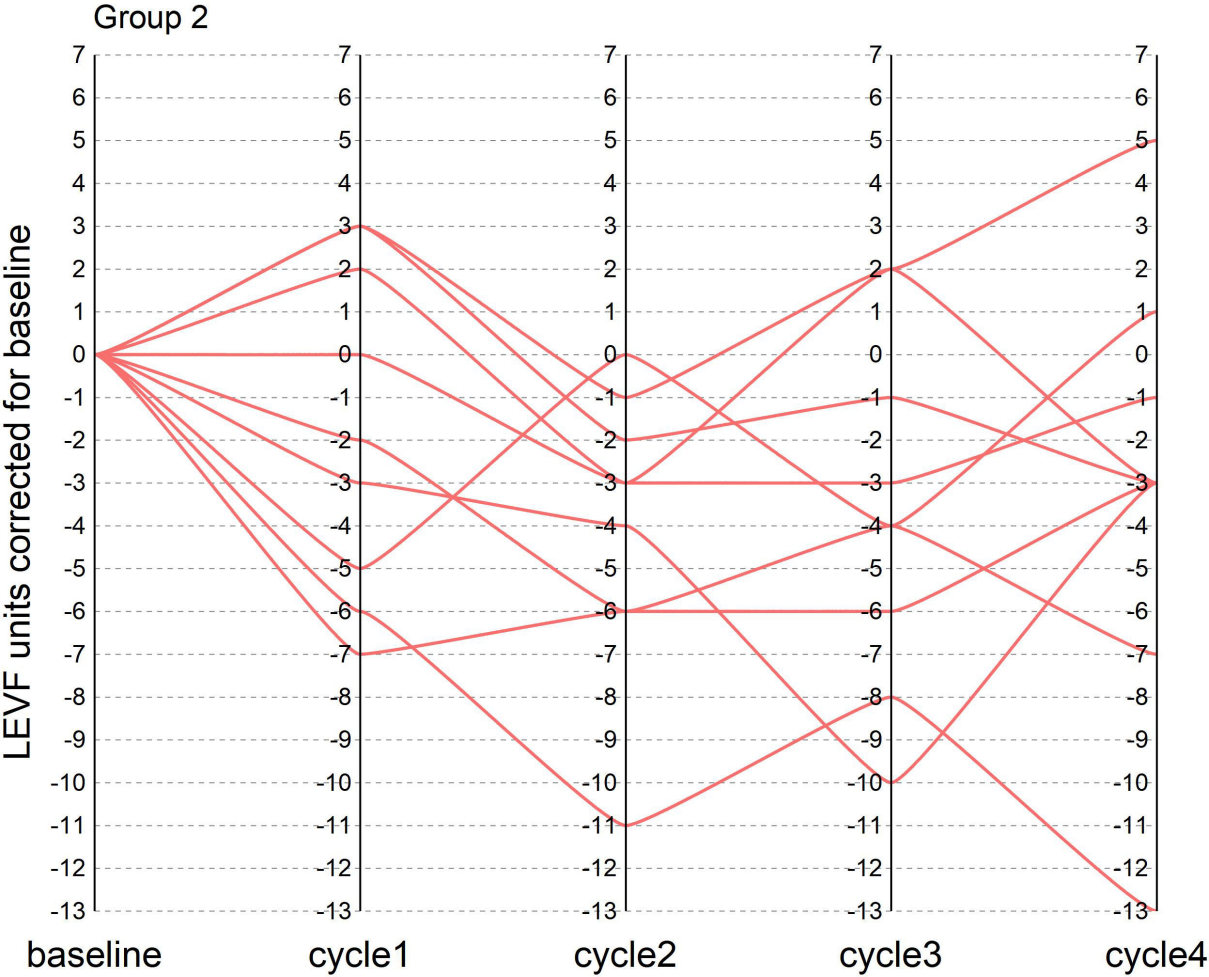
**Figure 2** shows the change in LVEF from baseline in patients who did not achieve tpCR during the treatment in our research.

During the course of neoadjuvant therapy, the patients exhibited better cardiac function and no events, such as heart rate failure. The LVEF of the patients before enrollment was greater than 55%, with a mean value of 61%. The mean LVEF after each of the 4 cycles of neoadjuvant chemotherapy was 60%, 57%, 59% and 58%, respectively. The mean difference between LVEF and baseline for patients after each cycle was −1%, −4%, −2%, and −3%, respectively (one patient failed to arrive on time for cardiac function testing after the third cycle Because of the COVID-19 epidemic prevention and control policy, so the mean of the cardiac function indicator before and after the two cycles was taken). There were two patients with a decrease in cardiac function greater than 10% during the treatment period, with maximum decreases of 11% and 13%, respectively, but the patients did not experience cardiac failure and the treatment course was not interrupted. In this trial, patients did not experience heart failure during neoadjuvant therapy, and only two patients had left ventricular ejection fractions below 50% in the fourth cycle, 46% and 49%, respectively. In the NeoSphere study in the neoadjuvant receiving dual target plus docetaxel group, three patients had a 10–15% decrease in LVEF from baseline, reaching below 50%, but above 40%. In contrast, no patient in the PEONY study had an LVEF decline of more than 10% or less than 50%. **Figures 1**, **2** show the changes in the left ventricular ejection fraction (LVEF) in 28 patients during treatment. 

## 4 Discussion

The HER2 gene is overexpressed in some breast cancers as a tumor-associated antigen and is considered an important breast cancer marker and prognostic factor (18). In HER2 positive breast cancer, ERBB2 (HER2) gene amplification is the most obvious (19). Therefore, HER2-positive breast cancers are more difficult to treat clinically due to their high malignancy and aggressiveness. However, with the emergence of NAT, HER2-positive breast cancer patients have faced more opportunities. In early clinical treatment, NAT is used for locally advanced breast cancer patients (20). These patients present difficulties or an inability to remove their tumors, and NAT can significantly reduce tumor size and clinical staging, which provides patients with the opportunity for surgical resection or breast preservation. With advancing research, evidence is supporting NAT for use not only for patients with advanced breast cancer, but also for patients with early breast cancer (21). The application of neoadjuvant therapy is becoming increasingly widespread. Firstly, patients who were originally unable to undergo surgery can undergo NAT to reduce tumor size and phase, and patients who were originally unable to undergo surgery can undergo surgery. Second, NAT can allow patients who originally needed total or extensive resection to preserve their breast or reduce the resection range. Third, NAT can assess whether tumor patients are sensitive to certain drugs and provide a basis for further postoperative treatment. Therefore, HER2-positive breast cancer patients and triple-negative patients are recommended for neoadjuvant chemotherapy in some guidelines. Studies suggest that breast cancer patients with a better response to NAT have a significantly better disease-free survival after achieving pCR postoperatively, and pCR can be used as a valid predictor of overall survival and disease-free survival in breast cancer patients (22). In fact, the use of anti-HER2 targeted agents in NAT has improved the prognosis of HER2-positive breast cancer patients even more significantly (23). Trastuzumab is a humanized monoclonal antibody derived from recombinant DNA that blocks HER2 homodimerization with the autologous body by binding to the extracellular structural domain (24). The NOAH study showed that the anti-HER2 drug trastuzumab, a biologic drug, had a higher pCR rate than conventional drug therapy, while the difference in the rate of cardiovascular adverse events was not statistically significant (11). Pertuzumab, like trastuzumab, is a humanized recombinant monoclonal antibody that binds to an extracellular structural domain II. It inhibits the formation of heterodimers of HER2 with other receptors (24). Dual-target synergy inhibits the HER2 signaling pathway at its origin, resulting in a more potent tumor suppressive effect.

The NeoSphere study marked the beginning of the first application of PH dual-targeting for neoadjuvant therapy. In 2012, the primary findings of the NeoSphere study were published, demonstrating that PH dual-targeting plus docetaxel had the highest tpCR rate of any regimen (39.3%), as opposed to the previous H single-targeting plus docetaxel regimen of 21.5%. Thus, the US FDA accelerated the approval of PH dual-target plus chemotherapy for the indication of neoadjuvant therapy in HER2-positive early-stage breast cancer patients. In subsequent studies, neoadjuvant trastuzumab or pertuzumab plus chemotherapy regimens with tpCR or bpCR 40.9% to 63.6%, with neoadjuvant regimens of 4 to 8 cycles was reported (25–28). The PEONY clinical study focused on the Chinese population. In this study, the tpCR rate in the PH dual target plus docetaxel group also reached 39.3%, which was higher than the 21.8% rate in the trastuzumab plus docetaxel group.

Currently, the dual targets in neoadjuvant THP regimens involve mostly the original trastuzumab and pertuzumab. Although domestic trastuzumab biosimilars have been approved for use, there are few reports of domestic dual-target PH for the treatment of HER2-positive breast cancer. This study uses a domestic Chinese dual-target HP plus docetaxel regimen to treat patients with ER/PR-negative and HER2-positive breast cancer and compares outcomes of patients in the ER/PR-negative and HER2-positive groups with those of the NeoSphere and PEONY studies with neoadjuvant treatment using the THP regimen alone to explore the efficacy and safety of the neoadjuvant THP (docetaxel, trastuzumab biosimilar TQB211 plus pertuzumab biosimilar TQB2440) regimen for the treatment of ER/PR-negative and HER2-positive breast cancer, providing more reference trials for the dual-target regimen in China.

The primary endpoint was met in this trial. The tpCR rate was 67.9%, which was higher than that observed in both subgroups in the NeoSphere and PEONY trials. This may be due to the equally good therapeutic efficacy of the Chinese HP dual-target drugs. However, some patients in the trial were treated with domestic trastuzumab plus pertuzumab, which was not a full domestic HP dual target, there were discrepancies in the consistency of reporting drug efficacy. However, with the addition of the domestic HP dual target, the final patient tpCR rate was higher than the previous tpCR of the same subgroup of NeoSphere and PEONY, indicating at least to some extent that the efficacy of domestic dual-target HP is not inferior to that of foreign HP.

Due to the use of the chemotherapy drug docetaxel in the three investigations, neutropenia or leukopenia was observed as an adverse effect. This is consistent with the results of previous clinical trials, in which hematological toxicity and abnormal liver function were thought to be associated with chemotherapeutic agents, while diarrhea was thought to be associated with both HER2 targeted therapy and chemotherapy (29, 30). The rate of adverse responses was relatively high in this trial, with neutropenia and leukopenia having the highest rates at 75% and 82.1%, respectively. It has been speculated that this is because the docetaxel dose of 75 mg/m^2^ is not tolerated by the majority of the Chinese population. The most common grade 3 or 4 adverse reaction in the PEONY study was neutropenia, with an incidence of 38.7% (17). In this study, the incidence of neutropenia among grade 3 or higher adverse events was 35.7%, which was roughly the same as the incidence in PEONY and 45% higher than the incidence of neutropenia among grade 3 or higher adverse reactions in the NeoSphere study (group receiving the THP neoadjuvant chemotherapy regimen), which was slightly lower (16). Furthermore, the incidence of leukopenia among grade 3/4 and above adverse reactions in this study was 46.4%, which was twice as high as the 20.6% (incidence of leukopenia) in PENOY study (17) and higher than the incidence in the THP group in NeoSphere (5%). Among other adverse effects, there was a higher incidence of diarrhea because pertuzumab blocked the formation of heterodimers responsible for signaling through EGFR and HER2, as also seen in the NeoSphere and PEONY studies.

Cardiac safety is also an important consideration when using dual-targeted regimens during neoadjuvant therapy. In this trial, none of the patients experienced heart failure during neoadjuvant therapy, and only two patients had left ventricular ejection fraction (LVEF) below 50% at cycle 4, 46% and 49%, respectively. The same subgroup of 3 patients in the NeoSphere study reported a 10-15% decrease in LVEF from baseline and therefore below 50%. However, these 3 patients showed improvement in cardiac function in cycle 4, with LVEF decreasing by less than 10%. In contrast, no patient in the PEONY study had a decrease in LVEF of more than 10% or less than 50%.

The current research has some limitations. To more accurately illustrate the efficacy and safety of dual-target domestic chemotherapy regimens in neoadjuvant therapy, validation of data from a large multicenter sample is needed. A portion of the study patients received pertuzumab plus domestic trastuzumab combination chemotherapy, which could not fully reflect the efficacy of the domestic dual target. In addition, this study had a short observation period and no long-term survival follow-up data. Due to such good results, we will continue to conduct Phase III clinical trial in the future. In future research, we will expand the sample size and also include the indicator of event free survival (EFS) in the study.

## 5 Conclusions

Overall, the tpCR rate achieved in this study was relatively high, although some was attributed to treatment with pertuzumab. Nonetheless, the overall tpCR rate was higher than that of the previous THP regimen trials. If trastuzumab and pertuzumab are successfully marketed in China, they will provide more first-line targeted drugs for the treatment of patients with breast cancer in China and more treatment options for physicians.

## Data availability statement

The raw data supporting the conclusions of this article will be made available by the authors, without undue reservation.

## Ethics statement

The studies involving humans were approved by Ethics Committee of Xijing Hospital (XJLL-YS-20201038). The studies were conducted in accordance with the local legislation and institutional requirements. The participants provided their written informed consent to participate in this study.

## Author contributions

DX: Writing – original draft. Data curation, Writing – review & editing. JW: Data curation, Writing – review & editing. JY: Resources, Writing – review & editing. YY: Resources, Writing – review & editing. XW: Data curation, Writing – review & editing. JXY: Methodology, Writing – review & editing. HW: Formal Analysis, Writing – review & editing. XX: Conceptualization, Writing – review & editing. YL: Methodology, Writing – review & editing. LY: Data curation, Writing – review & editing. LW: Supervision, Writing – review & editing. YW: Formal analysis, Methodology. WM: Visualization, Writing – review & editing. NL: Conceptualization, Writing – review & editing.
